# The impact of autoimmune comorbidities on the onset attack recovery in adults with AQP4-NMOSD and MOGAD

**DOI:** 10.1007/s00415-025-13180-3

**Published:** 2025-06-10

**Authors:** Sara Samadzadeh, Fiona Chan, Anna Francis, Layana Sani, Friedemann Paul, Nasrin Asgari, M. Isabel Leite, Ruth Geraldes, Jacqueline Palace

**Affiliations:** 1 Nuffield Department of Clinical Neurosciences, University of Oxford, Oxford, UK; 2 Institute of Regional Health Research and, Institute of Molecular Medicine, University of Southern Denmark, Odense, Denmark; 3 Department of Neurology, The Center for Neurological Research, Slagelse Hospitals, Slagelse, Denmark; 4 Experimental and Clinical Research Center, Charité—Universitätsmedizin Berlin, corporate member of Freie Universität Berlin and Humboldt-Universität Zu Berlin, Berlin, Germany; 5 University of Sydney, Sydney, NSW Australia; 6 Department of Clinical Neurology, John Radcliffe Hospital, Oxford University Hospitals Trust, Oxford, UK; 7 Department of Neurology, Frimley Health NHS Foundation Trust, Frimley, UK

**Keywords:** Comorbidities, Autoimmune disorders, Aquaporin-4 antibody neuromyelitis optica spectrum disorder, Myelin oligodendrocyte glycoprotein antibody disease, Disability

## Abstract

**Background:**

Aquaporin-4 neuromyelitis optica spectrum disorder (AQP4-NMOSD) often coexists with other autoimmune diseases (AIDs), whereas such comorbidities are less common in myelin oligodendrocyte glycoprotein antibody disease (MOGAD). This study investigates the impact of additional AIDs on early relapse recovery and disability in patients with AQP4-NMOSD and MOGAD.

**Methods:**

This retrospective study included patients aged > 16 years with AQP4-NMOSD (*n* = 175) or MOGAD (*n* = 221), who were followed at a nationally commissioned Oxford service and categorized based on the presence of at least one AID. Outcomes included recovery from the onset attack, visual recovery after the first optic neuritis (ON) attack (≥ 6 months post attack), time to first relapse and time to death. Incomplete visual recovery was defined as visual acuity worse than LogMAR 0.1. Optical coherence tomography (OCT) assessed retinal nerve fiber layer thickness and ganglion cell-inner plexiform layer volume in a subset.

**Results:**

In the AQP4-NMOSD cohort, 28% (*n* = 49) had at least one AID, compared to 11.3% (*n* = 25) in the MOGAD cohort (*p* < 0.001), with thyroid disease constituting the majority of these cases in both groups. In MOGAD, the median age of first attack was significantly higher in the AID group (46 years; IQR: 35–56) than in the non-AID group (35 years; IQR: 28–47) (*p* = 0.004), a difference that was not observed in the AQP4-NMOSD cohort. In both the AQP4-NMOSD (*n* = 175) and the MOGAD (*n* = 221) cohorts, age was a significant predictor of outcome in univariate analyses (AQP4-NMOSD: OR = 0.96 per year, 95% CI: 0.94–0.98, *p* < 0.001; MOGAD: OR = 0.97 per year, 95% CI: 0.94–0.99, *p* = 0.008). No significant differences were observed in clinical or visual recovery rates between AID and non-AID patients in either cohort. There were no statistically significant differences observed between AID and non-AID cohorts for clinical or visual recovery outcomes. Similarly, AID status did not influence time to relapse (AQP4-NMOSD: HR = 1.0, 95% CI: 0.63–1.58, *p* = 0.99; MOGAD: HR = 0.78, 95% CI: 0.40–1.52, *p* = 0.47) or time to death (AQP4-NMOSD: HR = 0.5, 95% CI: 0.18–1.36, *p* = 0.28). OCT analysis revealed no significant differences in retinal parameters between AID and non-AID groups in both cohorts.

**Conclusions:**

Additional autoimmune diseases are unlikely to significantly affect clinical or visual outcomes in early attacks in patients with AQP4-NMOSD and MOGAD.

**Supplementary Information:**

The online version contains supplementary material available at 10.1007/s00415-025-13180-3.

## Introduction

Aquaporin-4 neuromyelitis optica spectrum disorder (NMOSD) is an autoimmune disorder of the central nervous system (CNS) that primarily targets astrocytes, leading to inflammation, demyelination [1, 2]. The majority of NMOSD cases are associated with immunoglobulin G autoantibodies against aquaporin-4 (AQP4). Myelin oligodendrocyte glycoprotein antibody-associated disease (MOGAD) represents a distinct pathology, targeting myelin oligodendrocyte glycoprotein and primarily inducing demyelination rather than astrocytic damage [1, 3]. Both AQP4-NMOSD and MOGAD frequently manifest as optic neuritis (ON), which can result in significant neuronal damage and blindness. Recurrent relapses in both diseases contribute significantly to disability, highlighting the importance of identifying modifiable factors for improving recovery [4]. Despite the similar severity during acute attacks, AQP4-NMOSD attacks tend to recover less well than those in MOGAD leading to more severe disability [4, 5].

AQP4-NMOSD often coexists with various autoimmune diseases (AID), particularly systemic conditions, such as Sjogren’s syndrome and systemic lupus erythematosus, as well as organ-specific autoimmune disorders like autoimmune thyroid disease and myasthenia gravis [6–14]. It is observed that individuals with AQP4-NMOSD may have other autoantibodies, even in the absence of a diagnosed AID [13, 15–17]. In contrast, AID appear less common in MOGAD, where the prevalence of autoimmunity is generally lower than in AQP4-NMOSD [18, 19]. However, MOGAD can be rarely associated with anti-*N*-methyl-D-aspartate-receptor (NMDAR) encephalitis [20]. Glial fibrillary acidic protein (GFAP) is an intracellular antigen, and GFAP antibodies has been reported in various conditions, including AQP4-NMOSD. Given its lack of specificity and likely role as a marker of immune activation, GFAP encephalitis is considered a syndrome rather than a distinct autoimmune disease.

Visual outcomes are critical in both AQP4-NMOSD and MOGAD, yet the Expanded Disability Status Scale (EDSS) does not accurately capture visual impairment [21]. To address this limitation, dedicated visual acuity measurements are supplemented by optical coherence tomography (OCT) parameters, including peripapillary retinal nerve fiber layer (pRNFL) and ganglion cell-inner plexiform layer (GCIPL) thickness. In AQP4-NMOSD, acute ON shows mild pRNFL swelling, followed by rapid GCIPL and RNFL thinning [22–25]. MOGAD, however, often presents with marked and intense pRNFL swelling in acute ON, frequently accompanied by good visual recovery, typically due to retrobulbar involvement, aiding in distinguishing it from NMOSD and multiple sclerosis (MS) [26–28].

Although coexisting AID have been reported in a few studies, they have not been specifically studied as an isolated predictive factor for outcomes [29–31]. The purpose of this study was to investigate how having one or more additional autoimmune comorbidities affects clinical recovery from the initial attack in AQP4-NMOSD and MOGAD, as well as visual recovery and OCT parameters following the first attack onset of ON, in a large national cohort.

## Methods

### Study design and participants

AQP4-NMOSD [32] and MOGAD [33] patients seen in the nationally commissioned NMOSD/MOGAD Oxford service up to November 2023 were included, with antibody testing for both AQP4 and MOG conducted using the Oxford live cell-based assays [34]. Patients were only eligible if aged ≥ 16 years to ensure appropriate age matching, given the higher prevalence of MOGAD and the rarity of AQP4-NMOSD in the pediatric cohort. The other inclusion criteria were available data on clinical recovery from the onset attack, visual acuity outcomes at least six months post initial ON attack, and AID status. This clinical cohort was assessed longitudinally by the same two NMOSD expert consultants, and OCT was performed at a single center using Spectralis machines (Heidelberg Engineering, Heidelberg, Germany), data were extracted by FC and SS. Written informed consent was obtained under ethics reference 21/SC/0353, and data were prospectively collected and retrospectively analyzed. Demographic data, including age, ethnicity, and other potential risk factors such as sex, and self-identified races as well as clinical data, including attack phenotype, clinical recovery from the onset attack and first optic neuritis attack, time to first relapse and time to death, were recorded.

### Clinical definitions and outcomes

The AID group, which comprises autoimmune conditions, is detailed in Table 1. Immunotherapy medication use was documented for all patients. In the AQP4-NMOSD cohort, one patient with rheumatoid arthritis was on methotrexate, and another with necrotizing myositis was on 20 mg of prednisolone at the time of their first NMOSD attack. The remaining patients in the AQP4-NMOSD cohort, as well as all patients in the MOGAD cohort, were not receiving any immunotherapy medication for AID.

**Table 1:** Details of autoimmune comorbidity in the two cohorts

MOGAD (25 AID) (27 occurrences)—(196 non-AID)	AQP4-NMOSD (49 AID) (63 occurrences)—(126 non-AID)
Organ-Specific AID (21 occurrences/ 19 patients):	Organ-specific AID (34 occurrences/ 23 patients):
Autoimmune thyroiditis (6 occurrences)	Autoimmune thyroiditis (18 occurrences)
Psoriasis (2 occurrences)	Myasthenia gravis (non-thymoma AChR-positive) (7 occurrences)
Pernicious anemia (2 occurrences)	Ulcerative colitis (1 occurrence)
Crohn’s disease (2 occurrences)	Graves’ disease (3 occurrences)
Coeliac disease (2 occurrences)	Pernicious anemia (2 occurrences)
Autoimmune encephalitis (NMDA) (1 occurrence)	Inflammatory bowel disease (1 occurrence)
Uveitis (including panuveitis, anterior uveitis, vitritis) (2 occurrences)	Vitiligo (1 occurrence)
Graves’ disease (1 occurrence)	Warm autoimmune hemolytic anemia (wAIHA) (1 occurrence)
Inflammatory bowel disease (1 occurrence)	Non-organ-specific AID (29 occurrences/ 26 patients):
Vitiligo (1 occurrence)	Sjogren’s syndrome (5 occurrences)
Primary biliary cholangitis (1 occurrence)	Systemic lupus erythematosus (SLE) (8 occurrences)
Non-organ-specific AID (6 occurrences/ 6 patients):	Rheumatoid arthritis (7 occurrences)
Rheumatoid arthritis (4 occurrences)	Sarcoidosis (3 occurrences)
Seronegative arthritis (1 occurrence)	Mixed connective tissue disease (1 occurrence)
Polymyalgia rheumatica (1 occurrence)	Behcet’s disease (1 occurrence)
	Necrotizing myositis (1 occurrence)
	Immune thrombocytopenic purpura (1 occurrence)
	Antiphospholipid syndrome (1 occurrence)
	MPO positive vasculitis (1 occurrence)

*MOGAD* myelin oligodendrocyte glycoprotein antibody-associated disease, *AQP4-NMOSD* aquaporin-4-positive antibody neuromyelitis optic spectrum disorder, *AID* autoimmune disorders

The primary outcomes were (a) clinical recovery from the onset attack, and (b) visual recovery following the first ON attack. Clinical recovery was prospectively assessed by one of the treating NMO consultants leading the national service (IL or JP) and categorized as ‘complete recovery’ (large improvement of functional deficit or full recovery to baseline function) or ‘residual disability (patients who did not meet the above definition for complete recovery) at least 6 months after the onset attack. ‘Incomplete visual recovery’ was defined as a visual acuity (VA) below LogMAR 0.1 (equivalent to Snellen 6/7.5) observed ≥ 6 months after onset of an ON attack. Patients who had another attack within these 6 months were excluded. For cases of simultaneous bilateral ON, the eye that was worse >= 6 months post-ON was included in the analysis. Patients with other ophthalmological pathology leading to impaired visual acuity were excluded (*n* = 3; 2 MOGAD, 1 AQP4-NMOSD) were excluded only from the visual recovery from the first ON and OCT analyses as assessed by an ophthalmological consultant working within the NMO service. These patients were not excluded from the rest of the study. The secondary outcome was time to relapse and time to death.

### Optical coherence tomography

A subgroup analysis included patients who underwent OCT in Oxford at least 6 months following their first unilateral ON episode, with no contralateral ON episodes occurring during this period (AQP4-NMOSD: pRNFL, 25 ON/43 non-ON eyes; GCIPL/TMV, 21 ON/40 non-ON eyes. MOGAD: pRNFL, 36 ON/44 non-ON eyes; GCIPL/TMV, 33 ON/38 non-ON eyes.). All OCT scans were conducted at a single center using the Spectralis SD-OCT (Heidelberg Engineering, Germany, software V.6.16.6_INT). The OCT parameters analyzed included global peripapillary retinal nerve fiber layer (pRNFL) thickness, ganglion cell and inner plexiform layer (GCIPL) volume, and total macular volume (TMV). pRNFL thickness was measured using the Spectralis protocol with a 12° mm diameter ring around the optic nerve head. TMV was calculated through automated macular segmentation based on grid diameters of 1, 3, and 6 mm, cantered on the fovea and mapped to the nine ETDRS grid sectors. Scans were included only if they had a signal strength above 20 dB, were free of artifacts or retinal cystoid changes, met OSCAR-IB criteria [35, 36], and followed APOSTEL 2.0 reporting standards [37]. The exclusions were due to poor-quality OCT scans that prevented accurate segmentation of the retinal layers.

### Statistical analysis

Statistical analysis was performed in R (v4.3.1). Descriptive statistics were calculated using ‘base’ with ‘dplyr’ and ‘tidyr’ for data manipulation. Group comparisons utilized chi-square tests, with additional analyses by *t* tests and ANOVA from the ‘stats’ package. Significance was set at *p* < 0.05. Odds ratios with 95% confidence intervals were used to express clinical and visual recovery outcomes. In multivariate analyses, variables with univariate significance at *p* ≤ 0.1 were considered, and AID status was retained in all models regardless of significance. Only age was significantly associated with visual recovery and was therefore included as a covariate. To avoid overcrowding the models, additional univariate predictors, such as sex, baseline visual acuity, OCT measures, and time to relapse, were excluded as they did not show significant associations in univariate analyses. Onset phenotype and race were not included as the former was addressed separately in the ON subgroup analysis to account for attack phenotype, and the latter has not been shown to influence outcomes a priori. Time to relapse was analyzed using Kaplan–Meier survival curves (‘survival’ package). Differences were assessed with the log-rank test. Linear mixed-effects models using ‘nlme’ evaluated AID effects on OCT parameters, incorporating random effects. This study was exploratory without sample size calculation or multiple comparison adjustments.

## Results

### Autoimmune comorbidities in MOGAD and AQP4-NMOSD

Demographic and clinical characteristics are provided in Table 2 and Supplementary Tables 1, 2, and 3. A total of 396 patients were included (AQP4-NMOSD: *n* = 175; MOGAD: *n* = 221).

**Table 2:** Demographics and clinical characteristics

	MOGAD				AQP4-NMOSD			
	Total (221)	AID (25)	non-AID (196)	*P* value	Total (175)	AID (49)	non-AID (126)	*P* value
Age at onset (years) median (range)	35 (28–49)	46 (35–56)	35 (28–47)	0.004*	48 (35–59)	49 (38–60)	47 (35–59)	0.31
Age at ON event (years) median (range)	36 (28–48)	47 (36–59)	35 (28–46)	0.002*	44 (35–53)	43 (35–57)	45 (35–51)	0.53
Female *n* (%)	132 (59.7%)	19 (76.0%)	113 (57.7%)	*X*^2^(1) = 2.39, *p* = 0.12	145 (83.3%)	44 (89.8%)	101 (80.2%)	*X*^2^(1) = 2.54, *p* = 0.11
Ethnicity								
White	155 (70.1%)	17 (68%)	138 (70.4%)	–	93 (53.1%)	31 (63.3%)	62 (49.2%)	–
Asian	14 (6.3%)	3 (12%)	11 (5.6%)		22 (12.6%)	5 (10.2%)	17 (13.5%)	
Black-Caribbean	5 (2.3%)	1 (4%)	4 (2%)		36 (20.6%)	6 (12.2%)	30 (23.8%)	
Other/mixed	6 (2.7%)	0 (0%)	8 (4.1%)		10 (5.7%)	4 (8.2%)	8 (6.3%)	
Unknown	41 (18.6%)	4 (16%)	32 (16.3%)		14 (8.0%)	3 (6.1%)	9 (7.1%)	
Acute treatment *n* (%)								
Treatment received	179 (81.0%)	21 (84%)	157 (80.1%)	–	118 (67.4%)	32 (65.3%)	86 (68.3%)	–
No treatment received	41 (18.6%)	4 (16%)	37 (18.9%)		32 (18.3%)	11 (22.4%)	21 (16.7%)	
Data not available	1 (0.5%)	0 (0%)	1 (0.5%)		25 (14.3%)	0 (0%)	0 (0%)	
Recovery from onset attack *n* (%)								
Complete recovery	160 (72.4%)	18 (72%)**	142 (72.4%)	*X*^2^(1) = 0.00, *p* = 1	73 (41.7%)	23 (46.9%)**	50 (39.7%)	*X*^2^(1) = 0.495, *p* = 0.48
Residual disability	61 (27.6%)	7 (28%)	54 (27.6%)		102 (58.3%)	26 (53.1%)	76 (60.3%)	
Visual disability *n* (%)^#^								
Complete recovery	127 (76.1%)	14 (66.7%)**	113 (77.4%)	*X*^2^(1) = 0.65, *p* = 0.42	30 (38.9%)	12 (44.4%)**	18 (36%)	*X*^2^(1) = 0.23, *p* = 0.63
Incomplete recovery	40 (24.0%)	7 (33.4%)	33 (22.6%)		47 (61.1%)	15 (55.6%)	32 (64%)	

Data presented as median (range) or *n* (%). *p* values are from ANOVA analysis, and chi-square test as appropriate

*MOGAD* myelin oligodendrocyte glycoprotein antibody-associated disease, *AQP4-NMOSD* aquaporin-4-positive antibody neuromyelitis optic spectrum disorder, *AID* autoimmune disorders

^#^“Incomplete visual recovery” was defined as VA worse than LogMAR 0.1 (Snellen 6/7.5) ≥ 6 months post-ON. The worse eye was analyzed in bilateral cases. Patients with prior eye conditions or AD-related ocular pathology were excluded

In the AQP4-NMOSD cohort, 28% (*n* = 49) had at least one AID compared to 11.3% (*n* = 25) in the MOGAD cohort (*χ*^2^(1) = 16.82, *p* < 0.001). AIDs are listed in Table 1.

In the AQP4-NMOSD cohort, the most common AIDs observed were autoimmune thyroiditis (*n* = 18), followed by systemic lupus erythematosus (*n* = 8), myasthenia gravis (*n* = 7), and rheumatoid arthritis (*n* = 7), and Sjögren’s syndrome (*n* = 5). There was no significant difference between the AID and non-AID groups in terms of median age or sex ratio. More details on the demographic and clinical characteristics are provided according to AID categories as shown in Table 1.

In the MOGAD cohort, the most common AID observed was autoimmune thyroiditis (*n* = 6), followed by rheumatoid arthritis (*n* = 4). The median age of the first MOGAD attack was 35 years (IQR: 28–49), and was significantly higher in the AID group (46 years; IQR: 35–56) than in the non-AID group (35 years; IQR: 28–47) (*p* = 0.004). However, the proportion of females did not significantly differ between the AID group the non-AID group.

Among patients with AID, the timing of AID diagnosis was known in 20 MOGAD patients and 48 AQP4-NMOSD patients. Of those with known timing, 14 MOGAD patients (70%) were diagnosed with AID before the first attack and 6 (30%) after disease onset. Similarly, in the AQP4-NMOSD group, 34 patients (70.8%) were diagnosed with AID before the first attack and 14 (29.2%) after the onset. The timing of AID diagnosis was unknown for 5 MOGAD patients (20%) and 1 AQP4-NMOSD patient (2%).

### Effect of AID on clinical recovery from onset attack

**Fig. 1:**
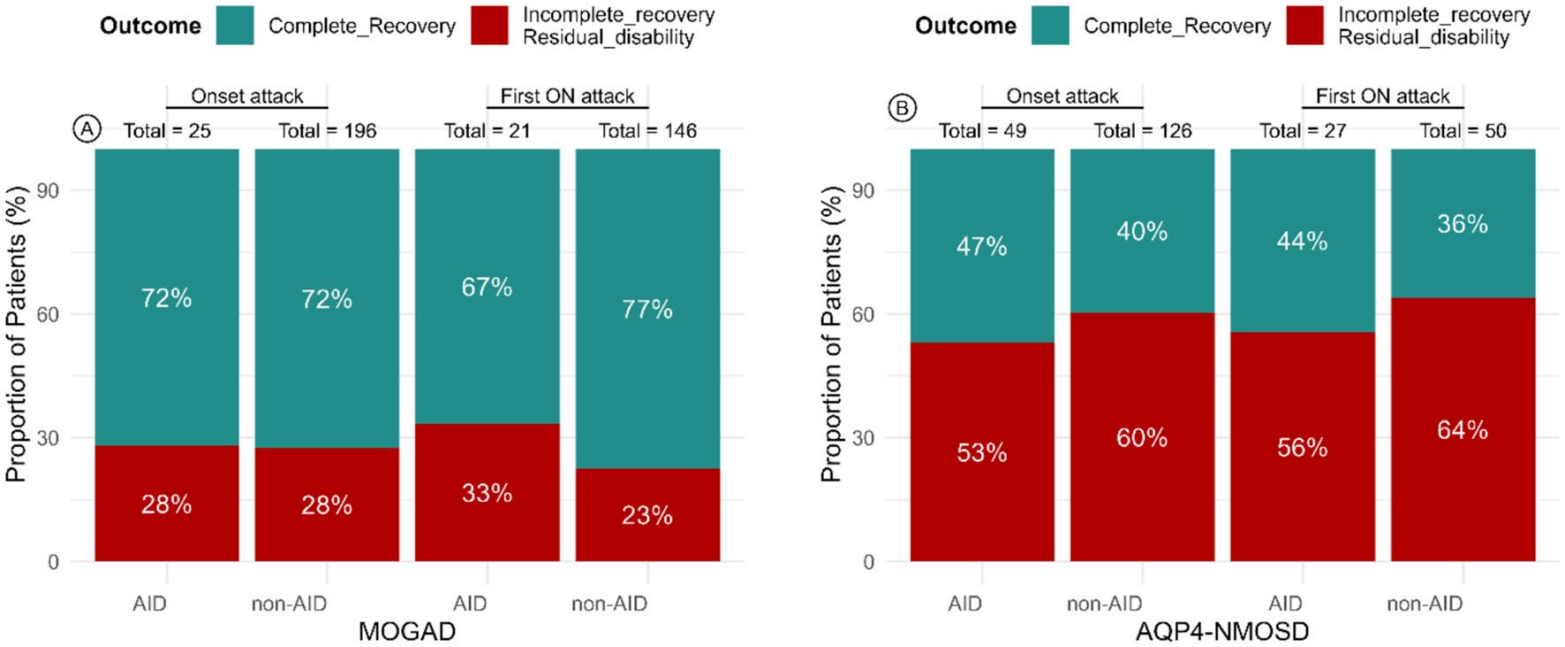
Percentage of recovery from onset attack or ON attack: complete recovery (green) or incomplete recovery/residual disability (red), MOGAD (**A**) and AQP4-NMOSD cohort (**B**). AQP4-NMOSD: Aquaporin-4 neuromyelitis optica spectrum disorder; MOGAD: Myelin oligodendrocyte glycoprotein antibody-associated disease; AID: autoimmune disorders; ON: optic neuritis

Clinical recovery rates from the onset attacks were similar in those with AID and those without in both the MOGAD and the AQP4-NMOSD cohorts. In the AQP4-NMOSD cohort (*n* = 175), complete recovery occurred in 46.9% (23/49) of the AID group and in 39.7% (50/126) of the non-AID group (OR = 1.33, 95% CI: 0.69–2.62, *p* = 0.38). In the MOGAD cohort, complete recovery occurred in 72% (18/25) of the AID group and in 72.4% (142/196) in the non-AID group (OR = 0.98, 95% CI: 0.40–2.63, *p* = 0.96) (Fig. 1; Table 3).

**Table 3:** Multivariate model of the effect of autoimmune disorders status on recovery

		MOGAD			AQP4-NMOSD		
		OR	95% CI	*P* value	OR	95% CI	*P* value
Recovery from onset attack	AID	0.98	0.4–2.63	0.96	1.33	0.69–2.62	0.38
	Age (year)	0.97	0.94–0.99	0.008*	0.96	0.94–0.98	0.0004*
	AID	1.36	0.51–4.17	0.56	1.5	0.73–3.00	0.26
	Age (year)	0.97	0.94–0.99	0.007*	0.96	0.94–0.98	0.0003*
Recovery from first ON attack	AID	0.58	0.22–1.64	0.29	1.42	0.54–3.71	0.47
	Age (year)	0.95	0.92–0.98	0.001*	0.97	0.93–1.0	0.08
	AID	0.93	0.25–2.95	0.91	0.62	0.23–1.67	0.34
	Age (year)	1.05	1.02–1.08	0.002*	1.03	1.0–1.07	0.06

The OR column represents odds ratio of incomplete recovery from onset attack and visual disability from first optic neuritis attack

*MOGAD* myelin oligodendrocyte glycoprotein antibody-associated disease, *AQP4-NMOSD* aquaporin-4-positive antibody neuromyelitis optic spectrum disorder, *AID* autoimmune disorders, *ON* optic neuritis

In both the MOGAD (*n* = 221) and the AQP4-NMOSD (*n* = 175) cohorts, age was a significant predictor of outcome in univariate analyses (MOGAD: OR = 0.97 per year, 95% CI: 0.94–0.99, *p* = 0.008; AQP4-NMOSD: OR = 0.96 per year, 95% CI: 0.94–0.98, *p* < 0.001). Therefore, age and AID status were included in the multivariate analyses for both cohorts. In these multivariate models, only age remained significantly associated with recovery, while AID status was not a significant predictor (Table 3).

To further refine the analysis, we conducted a subgroup analysis excluding organ-specific AIDs (specifically thyroid disorders), comparing outcomes between patients with no AID and those with non-organ-specific AIDs. Despite the limited sample size, no significant effect on clinical recovery from the onset attack was observed (Supplementary Table 4).

### Effect of AID on visual disability

In the AQP4-NMOSD cohort, the rates of incomplete visual recovery were higher than those observed in the MOGAD cohort, but they remained consistent between the AID and non-AID groups (55.6%, 15/49 vs. 64%, 32/126). Similarly, in the MOGAD cohort, the rates of incomplete visual recovery were comparable between the AID and the non-AID groups (33.4%, 7/25 vs. 22.6%, 33/196). The odds ratio (OR) for complete (or near complete) visual recovery in AID versus non-AID was 1.42 (95% CI: 0.54–3.71, *p* = 0.47) for AQP4-NMOSD and 0.58 for MOGAD (95% CI: 0.22–1.64, *p* = 0.29) (Fig. 1; Table 3).

In the AQP4-NMOSD cohort, there was a trend toward incomplete recovery with increasing age in the univariate analysis (OR = 0.97 per year, 95% CI: 0.93–1.0, *p* = 0.08), whereas in the MOGAD cohort, age was a significant predictor (OR = 0.95 per year, 95% CI: 0.92–0.98, *p* = 0.001). Therefore, age was included in the multivariate analysis for both cohorts. In the multivariate analyses, age remained the only significant predictor of visual recovery in the MOGAD cohort (OR = 0.95 per year, 95% CI: 0.92–0.98, *p* = 0.002), whereas in the AQP4-NMOSD cohort, neither age nor AID were significant predictors of poor outcomes (Fig. 1; Table 3).

The same analysis comparing patients non-AID to those with non-organ-specific AIDs showed no change in the results regarding visual disability (Supplementary Table 4).

### Effect of AID on time to first optic neuritis relapse

**Fig. 2:**
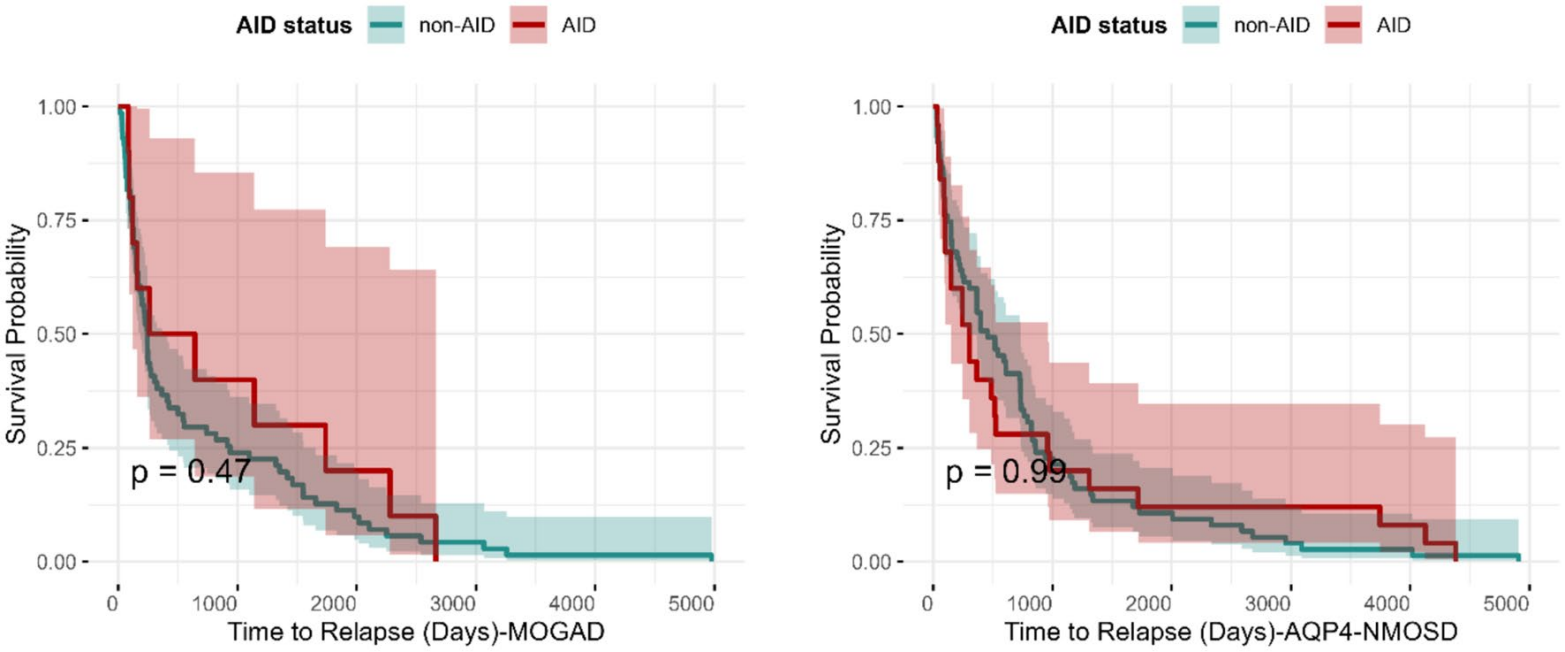
Kaplan–Meier survival curves depicting time to relapse in patients with AQP4-NMOSD (right) and MOGAD (left) stratified by the presence of comorbid AID. AID status is shown in red for non-AID and green for AID. Survival probability over time is displayed, with the p values indicating the statistical comparison between the two groups (*p* = 0.99 for AQP4-NMOSD and *p* = 0.47 for MOGAD). The shaded regions represent the 95% confidence intervals. MOGAD: Myelin oligodendrocyte glycoprotein antibody-associated disease; AQP4-NMOSD aquaporin-4-positive antibody neuromyelitis optic spectrum disorder; AID: autoimmune disorders

Figure 2 shows that no effect on time to relapse was seen in those who had AID compared with those without in the AQP4-NMOSD cohort (log-rank test HR 1.0 [95% CI: 0.63–1.58) *p* = 0.99] nor the MOGAD cohort [HR 0.78 (95% CI: 0.40–1.52) *p* = 0.47].

### Effect of AID on time to death

**Fig. 3:**
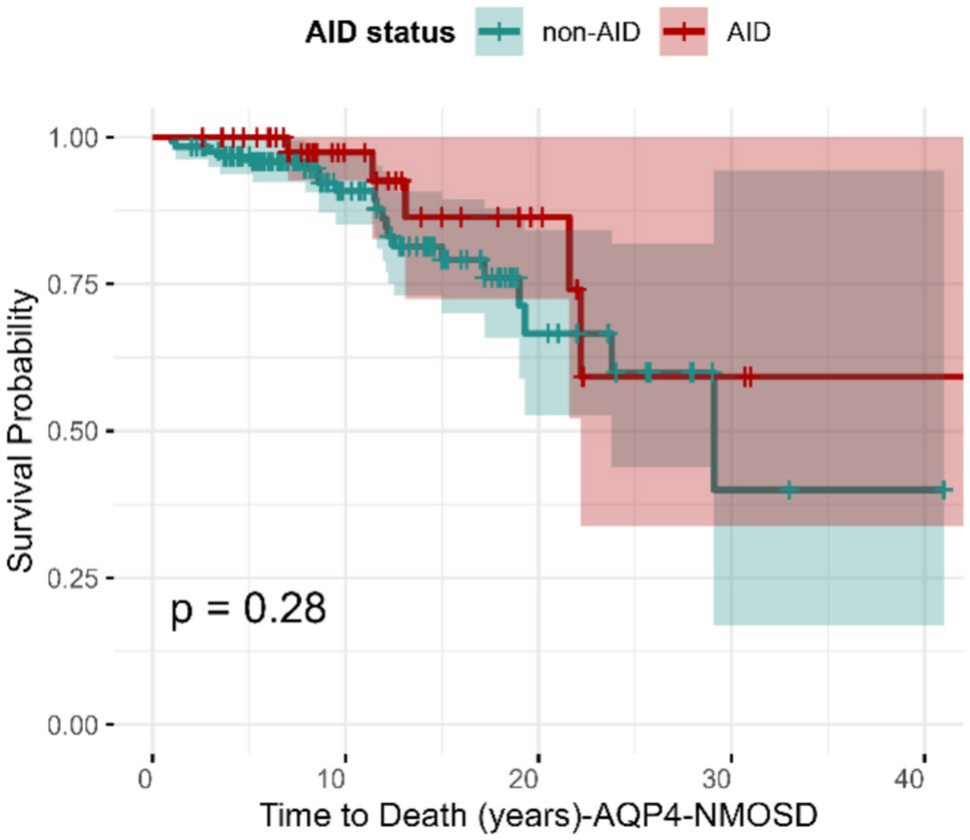
Kaplan–Meier survival curve depicting time to death in patients with AQP4-NMOSD, stratified by the presence of comorbid AID. AID status is shown in red for AID and green for non-AID. Survival probability over time is displayed, with a *p* value (*p* = 0.28) indicating no significant difference between the two groups. The shaded regions represent the 95% confidence intervals. AQP4-NMOSD: Aquaporin-4 antibody-positive neuromyelitis optica spectrum disorder; AID: autoimmune disorders

In the AQP4-NMOSD cohort (*N* = 175), patients with AID (6/49, 12.2% mortality) had fewer deaths compared to those without AID (29/126, 23.01% mortality). Time to death did not differ between those with and without AID, regardless of whether adjustments for age were made (HR 0.5, 95% CI: 0.18–1.36, *p* = 0.28) (Fig. 3). In the MOGAD cohort (*N* = 221), patients with AID (0/25, 0% mortality) experienced no deaths, while those without AID (1/196, 0.51% mortality) had one death. Given the low number of death events, a type II error cannot be excluded.

### OCT subgroup analysis

In the AQP4-NMOSD cohort, 25 eyes with ON and 43 eyes without ON were included for pRNFL analysis, while 21 eyes with ON and 40 eyes without ON were included for GCIPL and TMV analysis. In the MOGAD cohort, 36 eyes with ON and 44 eyes without ON were included for pRNFL analysis, while 33 eyes with ON and 38 eyes without ON were included for GCIPL and TMV analysis. Age, but not sex, was included in the analysis as it was a significant predictor of outcome. A linear mixed-effects model, with age included as a random factor, was used to evaluate the impact of AID status on pRNFL thickness, GCIPL volume, and TMV ≥ 6 months after the first ON attack.

**Fig. 4:**
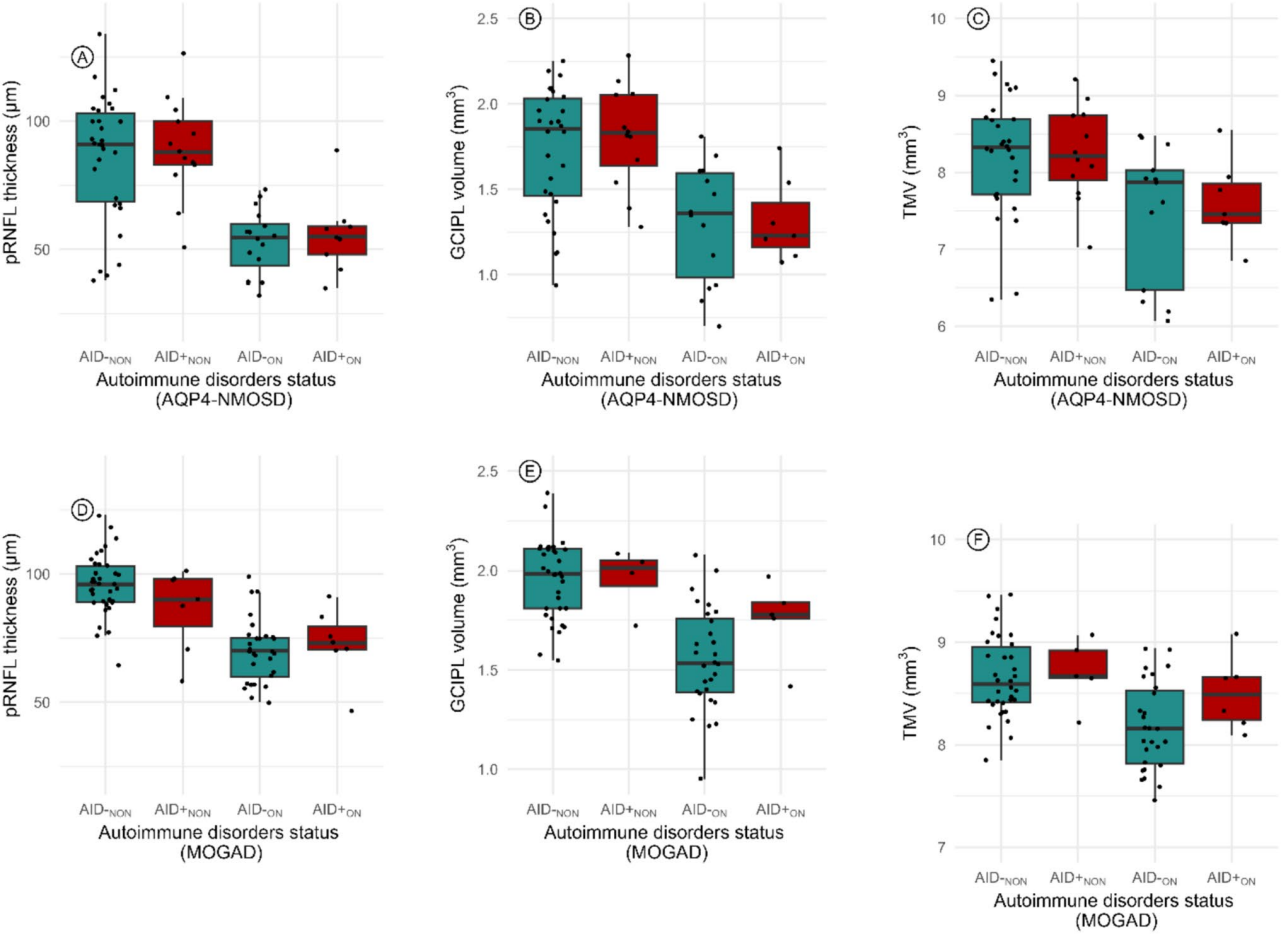
Impact of autoimmune disorders status on OCT parameters in AQP4-NMOSD and MOGAD Patients. **A** pRNFL thickness in AQP4-NMOSD patients by autoimmune disorders status; **B** GCIPL volume in AQP4-NMOSD patients by autoimmune disorders status; **C** TMV in AQP4-NMOSD patients by autoimmune disorders status; **D** pRNFL thickness in MOGAD patients by autoimmune disorders status; **E** GCIPL volume in MOGAD patients by autoimmune disorders status; **F** TMV in MOGAD patients by autoimmune disorders status. pRNFL: peripapillary retinal nerve fiber layer; GCIPL: ganglion cell-inner plexiform layer; TMV: total macular volume; AQP4-NMOSD: aquaporin-4-positive neuromyelitis optica spectrum disorder; MOGAD: myelin oligodendrocyte glycoprotein antibody disease; ON: optic neuritis; AID: autoimmune disorders. AID⁻NON: AD negative without optic neuritis; AID⁺NON: AD positive without optic neuritis; AID⁻ON: AD negative with optic neuritis; AD⁺ON: AD positive with optic neuritis; ns: not significant

In the AQP4-NMOSD cohort, AID status had no significant effect on any of the OCT measurements. In the MOGAD cohort, among eyes with no history of ON, a significant difference in pRNFL thickness was observed based on AID status. Eyes of patients with AID (*n* = 7) had significantly thinner pRNFL thickness (86.29 ± 16.03 μm) compared to eyes of patients without AID (*n* = 37) (96.00 ± 11.80 μm; Δ = − 9.71 μm) (*B* = − 13.1, SE = 5.93, *P* = 0.03). The model explained 11% of the variance marginally ($R_{m}^{2}$ = 0.11) and 45% conditionally ($R_{c}^{2}$ = 0.45) (Table 4 and Supplementary Table 5, Fig. 4). No significant differences were found in GCIPL volume or TMV, and no other OCT measurements differed between patients with and without AID.

**Table 4:** Impact of autoimmune disorders status on OCT parameters in AQP4-NMOSD and MOGAD patients

	With AD (no. of eyes)	Without AD (no. of eyes)	Absolute difference (μm, mean)	*B*	SE	*P* value	*R*^2^_marg_	*R*^2^_cond_
	pRNFL thickness							
	Thickness (μm, mean ± SD)							
AQP4-NMOSD with ON	55.67 ± 15.13 (9)	52.94 ± 12.64 (16)	2.73	2.79	5.80	0.63	0.04	0.88
MOGAD with ON	73.00 ± 13.67 (7)	69.97 ± 12.30 (29)	3.03	1.44	6.05	0.81	0.12	0.89

	GCIPL volume							
	Volume (mm^3^, mean ± SD)							
AQP4-NMOSD with ON	1.31 ± 0.24 (7)	1.31 ± 0.35 (14)	0.01	-0.015	0.14	0.92	0.16	0.90
MOGAD with ON	1.75 ± 0.20 (5)	1.56 ± 0.26 (28)	0.2	0.19	0.13	0.13	0.23	0.91

	Total macular volume							
	Volume (mm^3^, mean ± SD)							
AQP4-NMOSD with ON	7.61 ± 0.54 (7)	7.15 ± 1.23 (15)	0.46	0.36	0.42	0.41	0.33	0.92
MOGAD with ON	8.50 ± 0.36 (6)	8.09 ± 0.62 (28)	0.41	0.51	0.29	0.09	0.15	0.9

All *p* values > 0.05, indicating non-significance

*AQP4-NMOSD* Aquaporin-4-positive neuromyelitis optica spectrum disorder, *pRNFL* peripapillary retinal nerve fiber layer, *GCIPL* ganglion cell-inner plexiform layer, *TMV* total macular volume, *SD* standard deviation, *ON* optic neuritis, *AD* autoimmune disorders, *B* estimate, *SE* standard errors, *R*^*2*^* marginal *(*R*^2^_*marg*_) proportion of variance explained by the fixed factors alone; *R*^2^ *conditional* (*R*^2^_*cond*_) proportion of variance explained by both the fixed and random factors

## Discussion

Our large single-site study of nearly 400 AQP4-NMOSD and MOGAD patients showed no adverse effect of coexisting AID on recovery from the onset attack, first optic neuritis, or time to first relapse. This included clinical and visual assessment as well as OCT outcomes. Age however was a poor prognostic marker of outcome.

Consistent with previous studies, we observed a higher rate of AID in AQP4-NMOSD compared to MOGAD. The rates and the types of AID in both groups were similar to those reported in other studies. The prevalence of AID in MOGAD appears comparable to that of the general population (10.2%), whereas in AQP4-NMOSD, it is nearly three times higher [18, 19, 38, 39].

There is limited literature in this field. Although one study Lin et al. reported a higher relapse rate in the first year in NMOSD with AID than those without [30], another smaller study showed no difference in relapse rates over three years in AQP4-NMOSD patients with and without AID [29]. Another study of NMOSD patients rather than AQP4-NMOSD patients in a predominantly black population noted that those with AID had higher poor predictive factors, such as elevated baseline EDSS. However, both AID-NMOSD and NMOSD-only groups showed EDSS improvement after treatment, and although recovery was somewhat less pronounced in the AID group, it was still clinically meaningful. While the study did not adjust for covariates, the findings suggested that AID itself was not an independent predictor of poor outcome, indicating that overlapping AID may not substantially alter treatment response in NMOSD. [31]. The effects of AID on AQP4-NMOSD relapse recovery and in MOGAD as far as we are aware have not been published. Findings in MS remain inconsistent, with some studies indicating equivalent or slightly better outcomes in MS patients with AID [40–42], while others show worsened outcomes, particularly in radiological measures [43–45].

In summary, most of the previous studies suggest that the presence of comorbid AID does not necessarily correlate with worse long-term clinical outcomes in NMOSD patients. Our results contribute to this understanding by demonstrating that while AID is prevalent in AQP4-NMOSD and to a lesser extent in MOGAD, their presence does not adversely affect early clinical or visual outcomes in either condition.

Several factors may explain this neutral effect. First, AQP4-NMOSD and MOGAD are driven by specific autoantibodies targeting astrocytes and oligodendrocytes, respectively; therefore, their immune mechanisms and the subsequent clinical outcome may not be significantly influenced by an additional AID. Second, a major portion of the AID in this cohort is made up of thyroid disease and this is an easily treatable condition usually with T4 replacement. Also, patients with comorbid AID that significantly affect disability often receive immunotherapy medication that may also prevent attacks in AQP4-NMOSD or MOGAD, offsetting potential negative impacts. Third, the lack of shared antigens between these diseases and other AID likely does not results in minimal cross-reactivity and additional immune-mediated damage. It is also possible that patients with additional AID have a lower threshold for clinical manifestation and those without may have a higher threshold to trigger the clinical onset.

This study presents several limitations that should be considered when interpreting the findings. The samples size, particularly within subgroups, was relatively small for OCT analysis, which may reduce the statistical power to detect significant associations and limit the generalizability of the p-RNFL thickness findings in this subgroup. Additionally, visual acuity may be less sensitive than low contrast acuity in detecting deficits in optic neuritis; visual field assessments were not performed; and there is an inherent floor effect in OCT measurements, whereby patients with MOGAD and AQP4-NMOSD may exhibit severely thinned RNFL/GCIPL despite having contrasting visual acuity outcomes. Moreover, because the study only included patients aged > 16 years, the findings may not be representative of pediatric MOGAD cases, where the disease is more prevalent than MS. Potential confounding variables related to AID, including treatment regimens, disease duration, and severity, were not comprehensively collected or controlled for in the analysis, which could influence the observed associations. Furthermore, different autoimmune diseases may confer distinct relapse or disability risks; although grouping all autoimmune diseases was necessary given the small sample size, this may mask disease-specific effects. However, a subgroup analysis showed no difference when re-analyzing those with non-organ-specific AID or excluding thyroid AID patients. Additionally, patients with comorbid AID that significantly affect disability often receive immunotherapy, which may also reduce or prevent disability from AQP4-NMOSD or MOGAD attacks, potentially offsetting any negative effects. That said, pre-disease-onset immunotherapy use was rare in this cohort, occurring in only two AQP4-NMOSD patients and none with MOGAD. If the effect of AID on outcomes was small, it is possible that we were underpowered to detect it; however, the absence of hazard ratios above 1 suggests this is unlikely. A key strength of this study, however, is the homogeneity of the cohort, with all patients assessed uniformly at a single center, thereby eliminating site heterogeneity.

This large single study is the first to systematically examine the impact of autoimmune comorbid conditions on clinical outcomes in AQP4-NMOSD and MOGAD, showing no adverse effects not explainable by age on the early attacks. Further prospective studies are needed to evaluate longer-term outcomes, such as disability and relapse rates, within non-organ-specific AID subgroups of AQP4-NMOSD and MOGAD.

## Supplementary Information

Below is the link to the electronic supplementary material.

Supplementary file1 (DOCX 107 KB)

## Data Availability

The data that support the findings of this study are available from the corresponding author upon reasonable request. Access may be restricted to protect patient confidentiality or comply with institutional data sharing agreements.
