# Supplementary material for: The impact of autoimmune comorbidities on the onset attack recovery in adults with AQP4-NMOSD and MOGAD
Source: J Neurol. 2025 Jun 10;272(7):453. doi: 10.1007/s00415-025-13180-3 (PMC12152089; doi:10.1007/s00415-025-13180-3)
Supplement: Supplementary file 1 — Supplementary file1 (DOCX 107 KB) [file 415_2025_13180_MOESM1_ESM.docx]

| **Supplementary Table 1.** Onset clinical presentation in MOGAD and AQP4-NMOSD patients, stratified by presence or absence of autoimmune diseases (AID). | | | | |
| --- | --- | --- | --- | --- |
|  | MOGAD | | AQP4-NMOSD | |
|  | With AID  (25) | Without AID (196) | With AID  (49) | Without AID  (126) |
| Presentation of onset attack (No)  -Optic Neuritis  -Brain  -ADEM with encephalopathy  -ADEM without encephalopathy  -Transverse Myelitis  -Brainstem/Cerebellar  -Cortical/Cerebellar/Encephalitis  -Others | 19  3  0  0  3  2  1  2 | 145  5  1  4  14  1  0  7 | 23  3  0  0  2  3  0  7 | 42  9  0  0  9  10  0  7 |
| MOGAD: myelin oligodendrocyte glycoprotein antibody-associated disease; AQP4-NMOSD: aquaporin-4 positive antibody neuromyelitis optic spectrum disorder; AID: autoimmune disorders. | | | | |

| **Supplementary Table 2:** Interval Between Optic Neuritis and Visual Acuity Assessment by Diagnosis and AID Status (Time Difference in Months (Median [IQR]) | |
| --- | --- |
| Group | Diff (months) |
| AQP4-NMOSD without AID | 76 (32–151) |
| AQP4-NMOSD with AID | 83 (38–90) |
| MOGAD without AID | 67 (33–116) |
| MOGAD with AID | 88 (78–88) |
| MOGAD: myelin oligodendrocyte glycoprotein antibody-associated disease; AQP4-NMOSD: aquaporin-4 positive antibody neuromyelitis optic spectrum disorder; AID: autoimmune disorders | |

| **Supplementary Table 3**: Follow-up Duration and Statistical Comparison | | | |
| --- | --- | --- | --- |
| Group | N | Median (IQR) | p-value vs. Comparison Group |
| AQP4-NMOSD with AID | 49 | 15 (9–20) | p = 0.165 |
| AQP4-NMOSD without AID | 126 | 14 (9–18) |  |
| MOGAD with AID | 25 | 14 (5–16) | p = 0.000 |
| MOGAD without AID | 196 | 8 (5–11) |  |
| MOGAD: myelin oligodendrocyte glycoprotein antibody-associated disease; AQP4-NMOSD: aquaporin-4 positive antibody neuromyelitis optic spectrum disorder; AID: autoimmune disorders; IQR: Interquartile range | | | |

| **Supplementary Table 4:** Multivariate Model of the Effect of Non-organ-specific Autoimmune Disorders Status on Recovery | | | | | | | |
| --- | --- | --- | --- | --- | --- | --- | --- |
|  | | MOGAD | | | AQP4-NMOSD | | |
|  |  | OR | 95% CI | P-value | OR | 95% CI | P-value |
| Recovery from onset attack | Non-organ-specific AID | 0.99 | 0.56-1.85 | 0.97 | 1.07 | 0.66-1.72 | 0.78 |
|  | Age (year) | 0.97 | 0.94-0.99 | 0.005* | 0.96 | 0.94-0.98 | 0.0002* |
| Recovery from first ON attack | Non-organ-specific AID | 1.16 | 0.54-2.30 | 0.69 | 1.10 | 0.57-2.25 | 0.77 |
|  | Age (year) | 1.05 | 1.02-1.08 | 0.0005* | 1.03 | 1.0-1.07 | 0.16 |
| MOGAD: myelin oligodendrocyte glycoprotein antibody-associated disease; AQP4-NMOSD: aquaporin-4 positive antibody neuromyelitis optic spectrum disorder; AID: autoimmune disorders; ON: optic neuritis. The OR column represents odds ratio of incomplete recovery from onset attack and visual disability from first optic neuritis attack. | | | | | | | |

| **Supplementary Table 5:** Impact of Autoimmune Disorders Status on OCT Parameters in AQP4-NMOSD and MOGAD Patients | | | | | | | | |
| --- | --- | --- | --- | --- | --- | --- | --- | --- |
|  | With AD  (No. of eyes) | Without AD  (No. of eyes) | Absolute difference  (μm, mean) | B | SE | P value | R^2^_marg_ | R^2^_cond_ |
|  | pRNFL thickness | | | | | | | |
|  | Thickness (μm, mean ± SD) | |  | | | | | |
| AQP4-NMOSD without ON | 88.23 ± 19.14  (13) | 86 ± 24.48  (30) | 3.23 | 3.59 | 7.7 | 0.65 | 0.07 | 0.07 |
| MOGAD without ON | 86.29 ± 16.03  (7) | 96.00 ± 11.80  (37) | -9.71 | -13.1 | 5.93 | **0.03*** | 0.11 | 0.45 |
|  | GCIPL volume | | | | | | | |
|  | Volume (mm³, mean ± SD) | |  | | | | | |
| AQP4-NMOSD without ON | 1.81 ± 0.3  (12) | 1.73 ± 0.37  (28) | 0.08 | 0.05 | 0.12 | 0.71 | 0.14 | 0.15 |
| MOGAD without ON | 1.96 ± 0.17  (4) | 1.96 ± 0.20  (34) | 0 | 0 | 0.11 | 0.97 | 0.02 | 0.52 |
|  | Total macular volume | | | | | | | |
|  | Volume (mm³, mean ± SD) | |  | | | | | |
| AQP4-NMOSD without ON | 8.25 ± 0.62  (12) | 8.10 ± 0.99  (29) | 0.15 | 0.06 | 0.28 | 0.84 | 0.24 | 0.24 |
| MOGAD without ON | 8.71 ± 0.32  (5) | 8.66 ± 0.39  (34) | 0.05 | 0.05 | 0.2 | 0.79 | 0.02 | 0.68 |
| AQP4-NMOSD: Aquaporin-4 positive neuromyelitis optica spectrum disorder; pRNFL: Peripapillary retinal nerve fiber layer; GCIPL: Ganglion cell-inner plexiform layer; Total macular volume (TMV); SD: Standard deviation; ON: Optic neuritis; AD: autoimmune disorders. B: Estimate; SE: Standard errors; R^2^ marginal (R^2^ _marg_): Proportion of variance explained by the fixed factors alone; R^2^ conditional (R^2^ _cond_): Proportion of variance explained by both the fixed and random factors; All p-values > 0.05, indicating non-significance. | | | | | | | | |

**Definition of Visual Recovery and Justification of Threshold**

Visual recovery after optic neuritis (ON) was assessed based on visual acuity (VA) measured at least 6 months after the onset of the ON attack. Patients who experienced a second ON episode within this 6-month window were excluded from the analysis to avoid confounding by subsequent events. Poor visual recovery was defined as a VA below logMAR 0.1 (equivalent to Snellen 6/7.5), consistent with previous literature indicating that complete or near-complete recovery corresponds to logMAR 0.0 (Snellen 6/6) (Radner & Benesch, *Graefes Arch Clin Exp Ophthalmol*, 2019). The threshold was chosen based on prior analyses (doi: 10.1177/13524585251325069) that demonstrated its sensitivity in detecting associations with vascular comorbidities and smoking. Alternative thresholds were explored but did not meaningfully change the results. The distribution of VA scores (see Supplementary Figure) shows that logMAR 0.1 marks the inflection point between the bulk of patients with good recovery and the long tail of those with residual impairment.

| **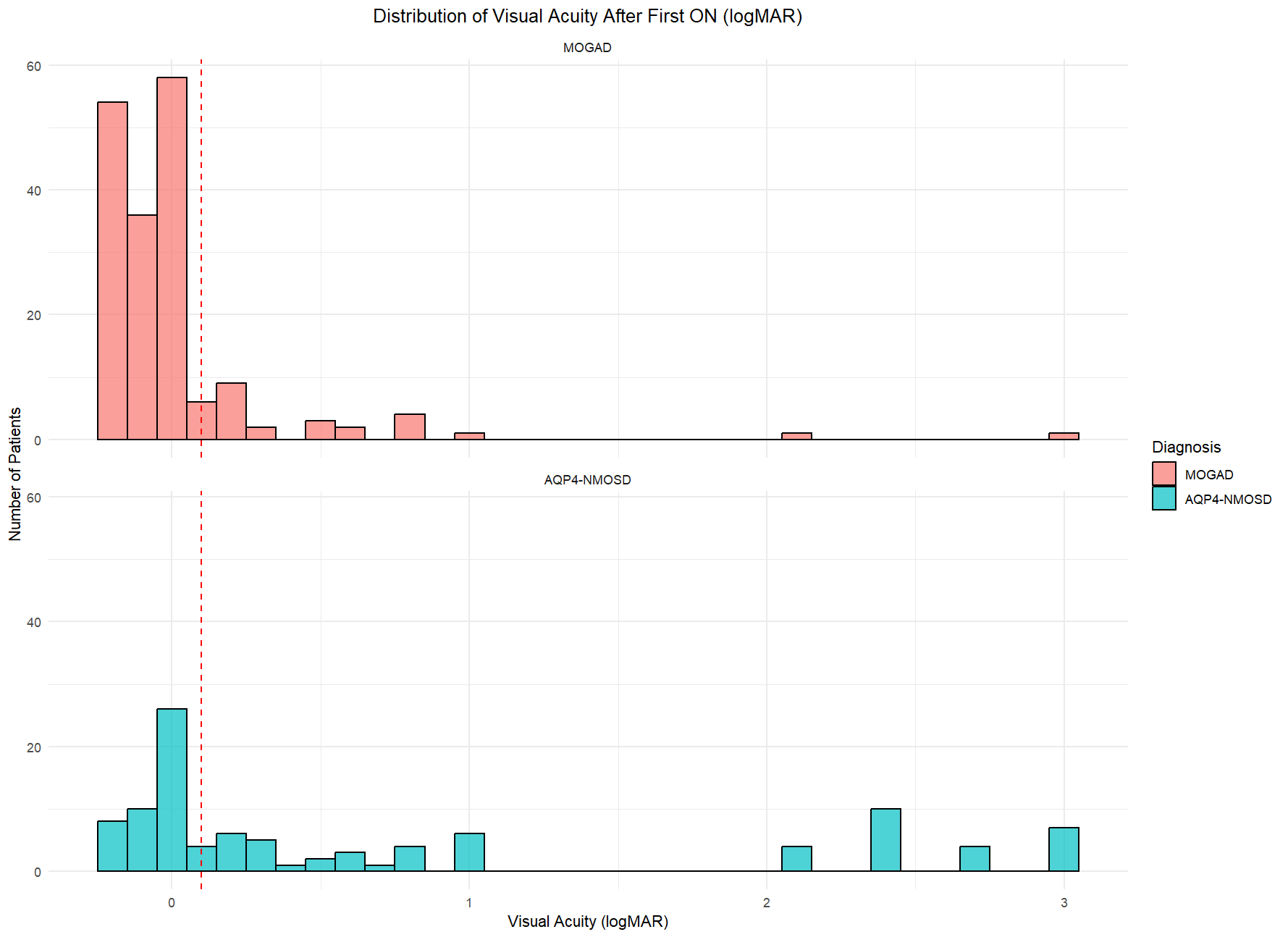** |
| --- |
| **Supplementary Figure 1:** Histogram of Visual Acuity (logMAR) After First ON by DiagnosisDistribution of visual acuity (logMAR) after the first optic neuritis (ON) episode in patients with myelin oligodendrocyte glycoprotein antibody-associated disease (MOGAD, top) and aquaporin-4 antibody neuromyelitis optica spectrum disorder (AQP4-NMOSD, bottom). The red dashed line represents the threshold of logMAR 0.1 (equivalent to Snellen 6/7.5), used to distinguish between complete and incomplete visual recovery. |
| 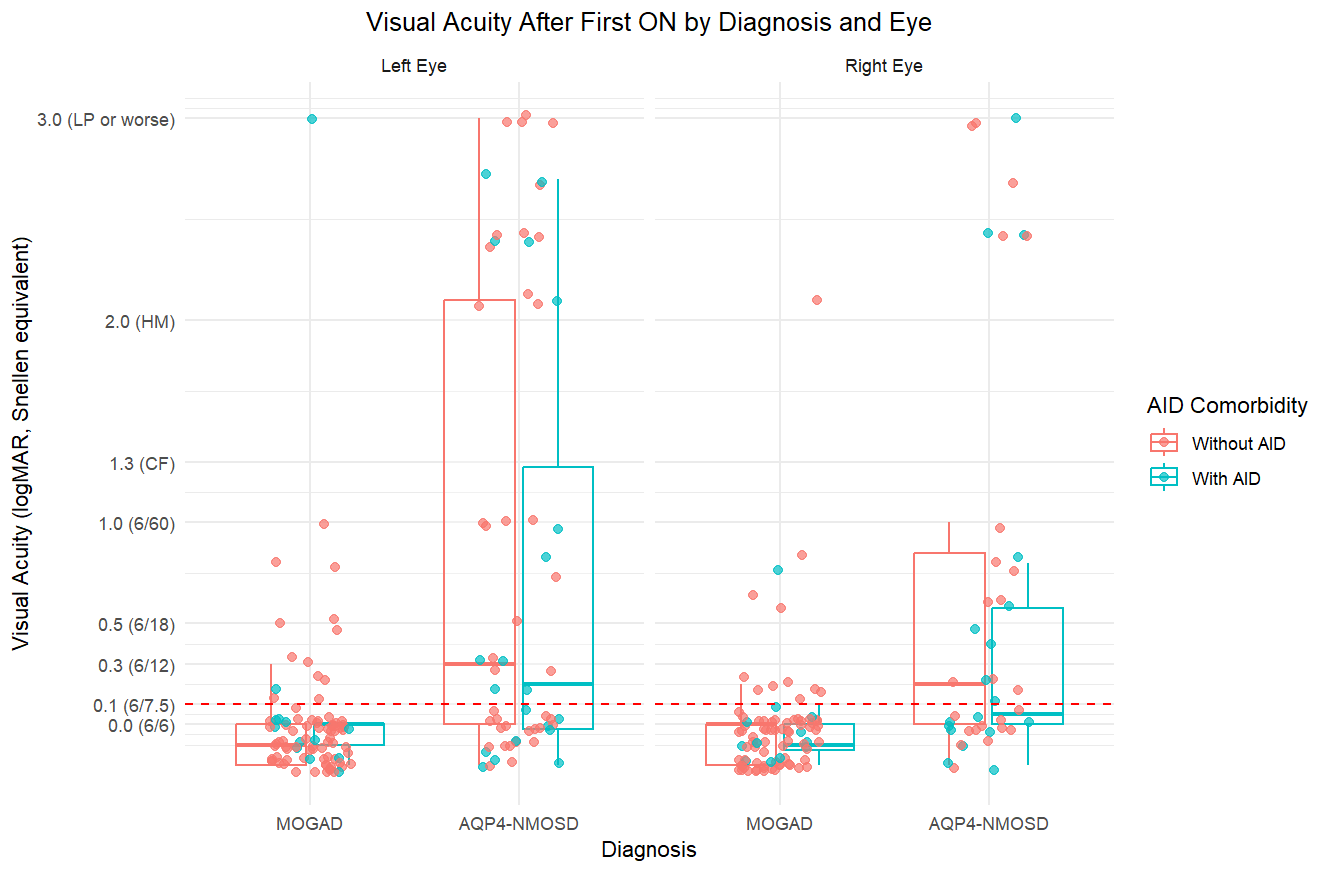 |
| **Supplementary Figure 2:** Boxplot of Visual Acuity by Diagnosis, Eye, and AID Comorbidity Visual acuity (logMAR) after first ON episode, shown separately for left and right eyes in MOGAD and AQP4-NMOSD, stratified by presence of autoimmune comorbidities (AID). The dashed red line indicates the threshold of logMAR 0.1 (Snellen 6/7.5).  Abbreviations: ON, optic neuritis; logMAR, logarithm of the minimum angle of resolution; AID, autoimmune disease; MOGAD, myelin oligodendrocyte glycoprotein antibody-associated disease; AQP4-NMOSD, aquaporin-4 antibody neuromyelitis optica spectrum disorder; CF, counting fingers; HM, hand motion; LP, light perception. |
